# The impact of extending nurse working hours on staff sickness absence: Evidence from a large mental health hospital in England

**DOI:** 10.1016/j.ijnurstu.2020.103611

**Published:** 2020-12

**Authors:** Idaira Rodriguez Santana, Misael Anaya Montes, Martin Chalkley, Rowena Jacobs, Tina Kowalski, Jane Suter

**Affiliations:** 1 Centre for Health Economics, University of York, Heslington, York, YO10 5DD, United Kingdom; 2 The York Management School, University of York, Freboys Lane, Heslington, York, YO10 5GD, United Kingdom

**Keywords:** shift patterns, mental health providers, nurses, sickness absence, health workforce, England

## Abstract

**Background:**

A pressing international concern is the issue of mental health workforce capacity, which is also of concern in England where staff attrition rates are significantly higher than in physical health services. Increasing demand for mental health services has led to severe financial pressures resulting in staff shortages, increased workloads, and work-related stress, with health care providers testing new models of care to reduce cost. Previous evidence suggests shift work can negatively affect health and wellbeing (increased accidents, fatigue, absenteeism) but can be perceived as beneficial by both employers and employees (fewer handovers, less overtime, cost savings).

**Objective:**

This study reports an evaluation of the impact of extending the shifts of nurses and health care assistants from 8 to 12 hours. Using data before and after the policy change, the effect of extended working hours on short term sickness (< 7 days) on staff is examined.

**Setting:**

The setting is six inpatient wards within a large mental health hospital in England where the shift extension took place between June and October 2017. The Data come from wards administrative records and the analysis is performed using weekly data (N=463).

**Methods:**

Causal inference methods (Interrupted Time Series and Difference-in-Difference) are used to compare staff sickness rates before and after the implementation, where the outcome variable is defined as the ratio of total sickness hours over the total scheduled working hours (full time equivalents) in a given week. Patient casemix, staff demographics, ward and time variables are included as controls.

**Results:**

Estimation results establish that the extended shifts are associated with an increased percentage of sickness hours per week of between 0.73% and 0.98%, the equivalent of a complete shift per week per ward.

**Conclusion:**

This is the first study to use causal inference to measure the impact of longer shifts on sickness absences for mental health workforce. The analysis is relevant to other providers which may increasingly look towards these shift patterns as a means of cost saving.

Contribution of the paper

## What is already known about the topic?

• There is a growing concern worldwide around mental health workforce capacity, characterized by staff shortages and high attrition rates and it has led providers to test new shift patterns to reduce costs.
• There is uncertainty regarding the benefits of the 12-hour shift system with perceived efficiencies from an employer perspective, which may be offset by adverse effects on employees’ health and wellbeing.

## What this paper adds

• It is the first to analyse the impact of 12-hour shifts on sickness absences for mental health wards in England.
• Relative to 8-hours, 12-hour shifts increase sickness absence between 0.73% and 0.98%
• The methodology employed allows identifying the effect of the 12-hour shifts on sickness absences from other factors, and the data used overcomes limitations of self-selection from previous studies where the policy has been optional.

## 1 Introduction

The mental health workforce faces all sorts of stressors; many of those are common to all health care staff, such as limited resources or overcrowded wards, whilst others are specific to the mental health care setting. Examples of the latter are: dealing with patients’ physical and/or verbal violent behaviour (Holloway et al., 2000, Renwick et al., 2016), the use of coercive measures such as restraint and detention of patients (Bonner et al., 2002), continuous monitoring of patients at risk of self-harm (Hagen et al., 2017) and in the most extreme cases with patients’ suicide (Fothergill et al., 2004) and with the public inquiries associated with these deaths, that often allocate blame to staff members (Holloway, Szmukler et al. 2000). According to O'Connor et al. (2018), the mental health workforce reports higher emotional exhaustion than emergency nurses and equal burnout to cancer professionals. The described stressors can explain, at least partially, the difficulties in recruiting and retaining mental health workforce. According to the World Health Organization (2007), there is a growing concern worldwide around mental health workforce capacity, characterized by staff shortages and high attrition rates. With excessive stress stemming from intense patient interactions over extended periods of time exposure to this working environment over a longer shift may have a greater impact on employee wellbeing (Edwards and Burnard, 2003).

Historically in England, mental health services have been chronically underfunded and, despite commitments to increase funding, providers are currently under financial constraints (British Medical Association, 2018). The latter, in addition to the described workforce pressures, have led mental health providers to test new models of delivering care to reduce costs (Kings Fund, 2015) (NHS Evidence, 2010). Increasing nurses’ shift length from 8 to 12 hours is becoming common practice in North America and the United Kingdom to address staff and financial pressures (Harris et al., 2015). The most recent estimates from Ball et al. (2015), based on Royal College of Nurses survey data, shows a substantial increase in the proportion of NHS nurses working 12-hour shifts; 31% in 2005 compared with 52% in 2009. The latter figure contrasts with the average for 12 European countries, also based on survey data, where only 14% of acute nurses were working 12 or more hours per shift (Griffiths et al., 2014).

There is uncertainty regarding the benefits of the 12-hour shift system with perceived efficiencies from an employer perspective, such as fewer handovers and less overlap between shifts (Dall' Ora et al., 2015); handovers and overlaps are regarded as unproductive and associated with a larger number of errors due to discontinuity of care (NHS Evidence, 2010). There are potential benefits to employees such as less travel time and longer periods between shifts (Knauth, 2007). However, these benefits may be potentially offset by stress and burnout (Stimpfel et al., 2012, Wallace et al., 2009), loss of job satisfaction, adverse effects on health and wellbeing, absenteeism and intention to leave (Dall' Ora et al., 2015, Dall'Ora et al., 2019).

There may also be adverse implications for the quality and safety of patient care associated with 12-hour shifts. Longer shifts have been associated with increased risk of making an error, e.g. not washing hands, not checking identity bracelets (Chudleigh et al., 2005, Ilhan et al., 2006) and decreased quality of care (Todd et al., 1989, Vik and MacKay, 1982). However, other studies have not found significant differences in terms of quality of care or number of incidents (Bloodworth et al., 2001, Stone et al., 2006). An analysis of the European RN4CAST survey, a multi-country cross-sectional nurse workforce study, shows that working 12-hour shifts is associated with higher odds of poor quality of care, greater risk of necessary nurse care left undone and higher odds of reporting being dissatisfied with their jobs (Griffiths et al., 2014). Nurses form the RN4CAST survey also reported that longer shifts reduced the opportunities to discuss patient care and to participate in continuing education activities (Dall'Ora et al., 2020).

There is a positive association between the amount of working hours and impairments to employees physical and mental health (Raediker et al., 2006). Previous studies, using cross-sectional and subjective nurse-reported data, have explored the effect of longer shifts on nurse health outcomes. Past findings show that nurses working longer shifts reported increased fatigue (Chen et al., 2014, Geiger-Brown et al., 2012, Lea and Bloodworth, 2002), greater stress burnout (Hoffman and Scott, 2003, Stimpfel et al., 2012) and higher risk of suffering work-related musculoskeletal disorders (Lipscomb et al., 2002, Trinkoff et al., 2006) all of which are likely to lead to increased sickness absence.

Sickness absence is a widely used outcome in health occupational research as it relates to workers’ social, economic and psychological processes (Merkus et al., 2012). Moreover, sickness absences are associated with several negative outcomes such as salary loss to the employee, salary costs of replacement staff, productivity losses and reduced quality of services.

This paper contributes to the literature by reporting an evaluation of the impact of a change from 8 to 12-hour shift patterns on sickness absences in a large National Health Service (NHS) mental health care provider in England by means of two causal inference methods: Interrupted Time Series and Difference-in-Difference. The aim is to identify whether there is a statistically significant association between longer shifts and sickness absence by comparing staff short-term sickness absence rates before and after the policy implementation. This is the second study, after Dall'Ora et al. (2019), that explores the association between longer shifts and sickness absences. These authors analyse longitudinal staffing data for 32 inpatient wards in England and find a significant association between longer shifts and increased sickness absences, however their analysis does not cover mental health staff. As nursing mental health patients can be physically and emotionally more demanding than nursing patients with physical health problems, the extension to 12-hour shifts in mental health wards might lead to worse staff outcomes than those observed for general inpatient wards.

## 2 Methods

### 2.1 Data

The subjects of this study are nurses and health care assistants from six geographically dispersed inpatient mental health wards from a large mental health NHS provider in England. The 12-hour shifts were introduced on three different dates: 1) Adult Mental Health Wards (wards A, B and C) on the 21st June 2017; 2) Older People Services Wards (wards D and E) on 18 September 2017 and 3) a Learning Disability Ward (ward F) on 16 October 2017. The data are a combination of roster records and routine hospital administrative data available before and after the introduction of the 12-hour shifts. The ward-level data includes information on daily staff sickness records, i.e. there is a record for each sickness absence registered, with each record containing information on the start date of the absence, the total number of sickness hours, the ward, and staff category. General ward information was available such as the total full time equivalent contracted staff per ward/month, information on patients’ casemix and staff demographics per ward/month. Daily data were converted to weekly data. There were a total of 463 observations, one per week and ward, and this data is distributed as follows: N  =  69 for ward A (Observations from Oct-16 to Jan-18); N = 73 for ward B (Oct-16 to Jan-18); N = 87 for ward C (from Jun-16 to Feb-18); N = 56 for ward D (from Feb-17 to Feb-18); N = 91 for ward E (from Jun-16 to Feb-18) and N = 87 for ward F (from Jun-16 to Jan-18).

### 2.2 Dependent variable: sickness absences

The duration of sickness absences ranges from 0 (where a sickness absence is reported but the staff is not on duty) to a maximum of 1912 hours (this is equivalent to a total of 239 8-hour shifts, and represents a long term sickness absence). As in Dall'Ora et al. (2019), a cut-off of 7 days is used to distinguish short and long term sickness absences. In this paper, given the limited time frame and number of observations after the implementation of the 12-hour shifts, the focus is on short-term sickness absences. This focus results in dropping 30% of individual observations for sickness absences before aggregating the sample to the ward / week level.

The outcome variable is defined as the ratio of the total sickness hours per ward/week and the total number of contracted hours per ward/week. The outcome measure is expressed as the percentage of total sickness hours per ward/week.

### 2.3 Control variables

Additional covariates are included to control for the effect that other factors can have on the outcome variable such as variations in patients’ severity and complexity of mental ill-health, and staff demographic characteristics, all measured at the week/ward level. The following control variables are included: the monthly average Health of the Nation Outcome Scale (HoNOS) score, a clinician-rated outcome measure capturing patient's severity at ward level. The Health of the Nation Outcome Scale score is a widely used instrument measuring symptom severity and social functioning and it ranges from 0 (best) to 48 (worst) (Jacobs, 2009). Mental health providers are required to categorise all patients into groups designed to capture similar levels of need, called clusters, which in turn are categorised into three superclusters (Moscelli et al., 2018) namely: 1) non-psychotic, 2) psychotic and 3) organic. In our sample, the majority of organic patients are in wards with only other organic patients, so in order to capture these groups, but avoid multicollinearity, we only include a dummy variable indicating the percentage of psychotic (category 2) patients per ward and per month.

The inclusion of control variables improves the estimation in the presence of time-varying confounders. Month dummies are included to capture variations in sickness absences that might be associated with seasonal factors (e.g. flu season). In order to account for workload pressures, a variable indicating the volume of patients is included, measured as the number of patients per month per ward. The total number of staff (headcount) per month is included as workload has been identified as a workplace stressor (Wallace et al., 2009). The percentage of registered nurses controls for different capacities of staff available per week due to qualification level. We control for various staff characteristics: average staff age, percentage of female staff and percentage of white staff as previous literature indicates that younger mental health staff suffer more from burnout, with mixed results by gender (Moore and Cooper, 1996), and that ethnic minority mental health staff can be exposed to additional stressful situations linked to cultural misunderstandings with patients, or to racist behaviours (Kavanagh, 1991).

### 2.4 Econometric strategy

Two different estimation approaches are followed: Interrupted Time Series and Difference-in-Differences. Both methods have been used extensively in observational studies to address causality (Angrist and Pischke, 2009, Cameron and Trivedi, 2005, Penfold and Zhang, 2013). For both estimation methods three model specifications are estimated: (1) that only includes the policy effect parameter and time dummies; (2) which includes all the variables in (1) plus patient casemix and (3) which includes all the variables in (2) plus staff demographic variables.

### 2.5 Interrupted Time Series

The Interrupted Time Series analysis uses data gathered before the implementation of the 12-hour shifts to forecast the trajectory of sickness absences, had the shift pattern change not come into practice, and compares it to the observed sickness absences to estimate the effect associated with the longer shifts. In the Interrupted Time Series analysis, the time of the implementation of the policy is standardised to zero, as if the policy change was implemented in all wards at the same time, and then all the data points observed before and after the policy are used to estimate an overall effect of the policy in all six wards.

In particular, the interest is in estimating the effect of the switch from 8 to 12-hour shifts on the percentage of sickness absences per week, so the equation of interest for wards j =1,..,J is estimated by the following specification:

(1) $$y_{jt}=\tau_{ITS}D_{j}+\delta D_{j}t+\alpha_{0}t+\alpha_{1}S+X_{jt}^{\prime }\beta +\gamma_{j}+\epsilon_{jt}$$

where *y_jt_* represents the percentage of sickness absences in ward j and week t; *D_j_* is a dummy variable that captures the switch to 12-hour shifts and takes the value 1 for those observations after the policy implementation and 0 for those observed before. *D_j_t* is the policy dummy interacted with trend, *t* captures the weekly trend, and a set of dummy variables S, captures monthly seasonality. *X_jt_* is a vector that represents patients’ characteristics and staff demographics per ward, *γ_j_* is the ward fixed effect and $\epsilon_{jt}$ the error term. The effect of the policy in this setting is measured by *τ_ITS_* (Cook et al., 2002).

The use of covariates in the interrupted time series requires that all covariates considered in *X_jt_* should be independent of the policy, in other words they should be stable and not affected by the introduction of the policy (Lee and Lemieux, 2010). This assumption for each variable is tested in a regression discontinuity framework (Calonico et al., 2019) and a failure of the assumption would invalidate the use of these covariates in the regression.

### 2.6 Differences in Differences

The Difference-in-Differences approach takes advantage of the different timings of introducing the policy, in this case the period after Adult Mental Health Wards (wards A, B and C) had experienced the introduction of 12-hour shifts and we use the data of the other three wards that were still operating under the 8-hour shift policy (wards D, E and F) as controls. Between June and September 2017, there were three wards affected by the policy and three unaffected which allows us to utilise data from the latter group to estimate what would have happened to the sickness absences of affected wards if the policy had not been implemented. The Difference-in-Differences approach is specified as:

(2) $$\begin{array}{ccc} y_{jt} & = & \theta Treated_{j}+\lambda time+\tau_{DID}Treated_{j}time+\alpha_{1}S\\ & & +\;X_{jt}^{\prime }\beta +\gamma_{j}+\epsilon_{jt} \end{array}$$

where *Treated* takes the value of 1 for the wards where the policy was implemented first, in this case wards A, B and C and zero for wards D, E and F. The variable *time* corresponds to zero before the introduction of the policy in the treated wards, in this case 21 June 2017, and one after this. Following the standard Difference-in-Differences approach, in this equation *τ_DID_* is the difference-in-difference estimation which corresponds to the iteration term between treated and time (Cameron and Trivedi, 2005). The sample was restricted to 18 September 2018 where the other three wards (D, E and F) served as controls, because wards D and E were also affected by the policy at that time.

The option of applying the Difference-in-Differences estimation to the period between the introduction of the policy in wards D, E and ward F was explored. However, this was not possible due to data limitations as there is only one month of difference between the implementation of the policy on these wards.

### 2.7 Robustness Checks

To test the robustness of the estimates, a series of checks were carried out: (i) A placebo test is used that assumes the introduction of an arbitrary extended shift policy on the same day and month, one year before. (ii) The regressions are fitted using pooled Ordinary Least Squared instead of fixed effect panel data and year and week dummies are added. (iii) The effect of the 12-hour shifts on alternative outcome variables are tested: longer sickness absences up to 14 days, and shorter absences up to 2 days. (iv) The policy implementation date is artificially moved up to six weeks prior to the introduction of the policy and up to six weeks after to test for anticipatory and delayed effects.

### 2.8 Ethical approval

This study received ethics approval from the Health Research Authority - 18-HRA-0454.

## 3 Results

### 3.1 Descriptive statistics

**Fig. 1:**
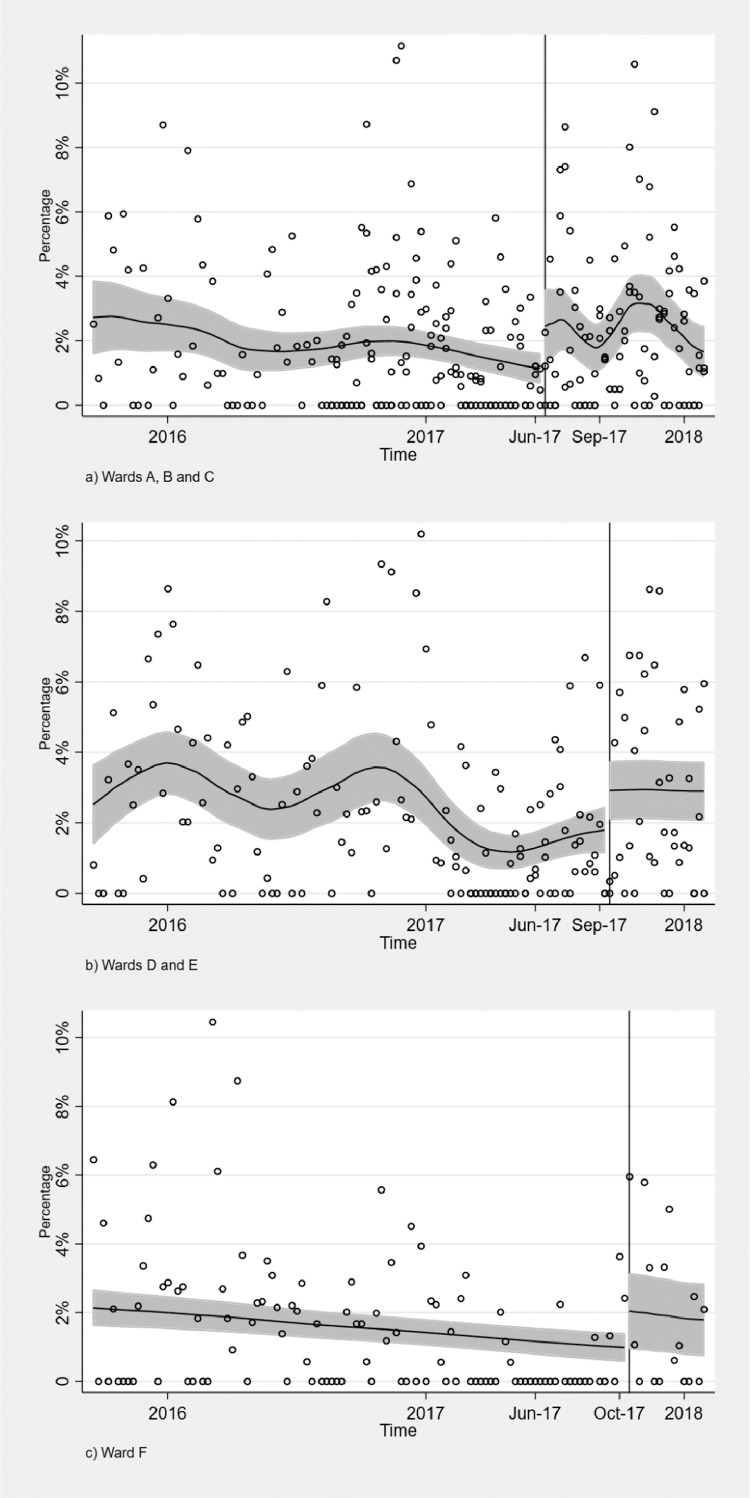
Change in policy timing per ward. The solid horizontal line is the local polynomial smooth of the dotted scatter values of percentage of hours of sickness absence up to 7 days using a triangle kernel function; the shaded area represents the 95% confidence interval around it and the vertical line indicates the timing of the introduction of the policy.

**Fig. 2:**
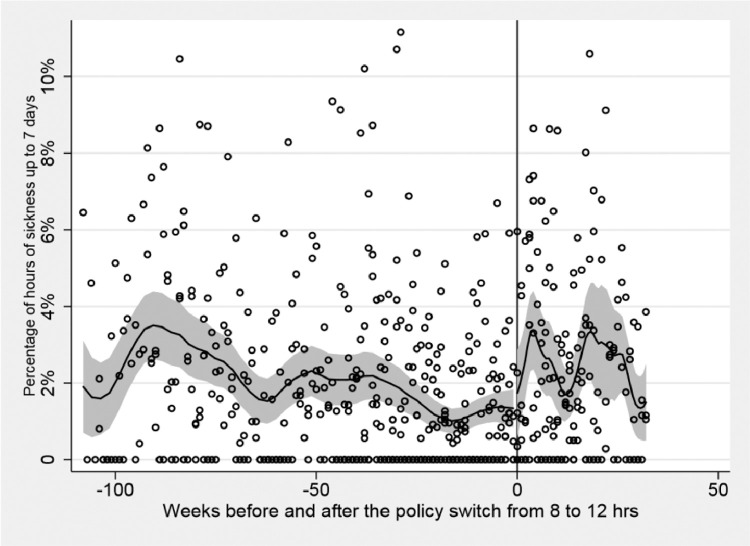
Sickness absence in percentage up to 7 days, before and after policy implementation. Policy timing cut-off standardised at time zero. The solid horizontal line is the local polynomial smooth of the dotted scatter values using a triangle kernel function; the shaded area represents the 95% confidence interval around it and the vertical line indicates the timing of the introduction of the policy.

Fig. 1 shows the change in the percentage of sickness absence before and after the policy introduction, grouped by type of wards, while Fig. 2 displays all wards grouped together, by standardising the timing of the introduction of the policy to zero. It shows the percentage of sickness absences per ward before and after the policy introduction. From a visual examination, it appears that the introduction of 12-hour shifts increased the percentage of sickness absences per week in all wards.

Table 1 shows the number of observations and the descriptive statistics for all the variables included in the analysis (means and standard deviation) for the overall sample (1) and for the observations before (2) and after (3) the introduction of 12-hour shifts. The difference in means (t-test) (4) between (2) and (3) for all the covariates is also included.

**Table 1:** Descriptive statistics before and after the introduction of the 12-hour shift policy (means and standard deviation in parentheses) and difference in means test.

	(1)	(2)	(3)	(4)
	Overall	Before	After	
	Mean (SD)	Mean (SD)	Mean (SD)	Diff. Means
Outcome variables sickness up to 7 days				
Sickness hours per week (Perc. x 100)	1.96	1.68	2.45	0.76***
	(2.26)	(2.12)	(2.43)	
Control variables				
Casemix adjustment monthly average HoNOS score	18.72	18.65	18.85	0.20
	(2.25)	(2.24)	(2.27)	
Supercluster group 2 (Perc. x 100)	43.75	42.20	46.48	4.28
	(33.73)	(34.06)	(33.06)	
Number of patients per month	11.08	10.86	11.47	0.61*
	(2.96)	(2.91)	(3.01)	
Staff average age (in years)	45.01	45.16	44.74	0.42
	(4.09)	(4.12)	(4.05)	
Female staff (Perc. x 100)	78.90	79.40	78.03	1.37*
	(6.48)	(5.40)	(7.98)	
Staff ethnicity white (Perc. x 100)	94.32	94.75	93.57	1.17**
	(4.63)	(4.75)	(4.30)	
Registered Nurses (Perc. x 100)	35.88	36.93	34.02	2.91***
	(6.80)	(7.05)	(5.93)	
Number of staff per month	30.58	31.35	29.21	2.14*
	(8.96)	(10.49)	(4.98)	
Total number of observations (week/ward)	463	296	167	463

*Note:* *<0.1, **<0.05, ***<0.01. Pooled descriptive statistics, the sample is divided between before and after by the time of the implementation of the 12-hour shift policy for each ward.

For the outcome variable, the average percentage of sickness absences in hours per week (first row, Table 1), before the implementation of the policy, is 1.68% whilst after, it is 2.45% resulting in an increase of 0.76%, statistically significant at the 99% confidence level. There are no statistically significant differences associated with patient casemix before and after the introduction of the policy. However, there are some differences in the staff demographic composition (sex, ethnicity) of the wards before and after the introduction of the 12-hour shifts. These differences might be linked to changes in the total number of staff per ward as the percentage of registered nurses per ward has decreased by nearly 3% following the introduction of longer shifts.

**Fig. 3:**
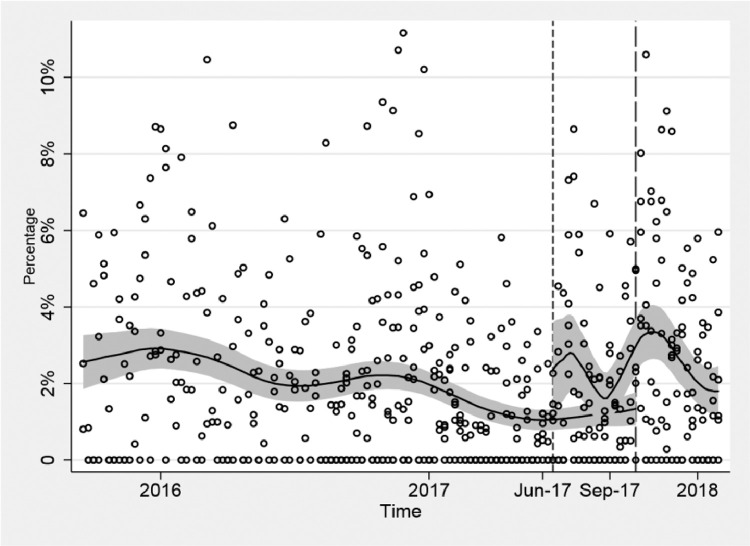
Difference-in-Differences identification strategy: The policy timing varies, three wards (A, B and C) introduced the 12-hour shifts in June, two in September (wards D and E) and one in October 2017 (ward F). Between June 2017 to September 2017 there are three wards affected by the policy and three wards unaffected by the policy. The solid horizontal line is the local polynomial smooth of the dotted scatter values using a triangle kernel function; the shaded area represents the 95% confidence interval around it and the vertical lines indicate the timing of the introduction of the policy.

Fig. 3 shows the difference in timing in the introduction of the policy, that justifies the Difference-in-Differences approach. In the intersection, the lower smoothed curve represents the wards (D,E and F) with delayed timing in the application of the policy, between June and October 2017, i.e. the control group, while the upper smoothed line represents the wards (A, B and C) with policy implementation in June 2017, i.e. the treatment group. The figure suggests that the wards in the control group maintained a more or less stable percentage of sickness absence, whereas in the treatment group the sickness absence increased.

### 3.2 Regression results

Table 2 displays the main estimation results. Columns (1)-(3) show the interrupted time series estimates whilst columns (4)-(6) present the difference-in-difference estimation results. The preferred specification includes all control variables given by column (3) for the Interrupted Time Series analysis and column (6) for the Difference-in-Differences analysis. According to the Interrupted Time Series results the switch to 12-hour shifts increased sickness absences by 0.7%, whilst Difference-in-Differences results estimates a slightly larger increase of approximately 1%. However, there is no statistically significant difference between both estimations according to their confidence intervals. For full results, refer to Appendix A.

**Table 2:** Interrupted time series (ITS) regression results and Difference-in-differences (DID) regression results.

	Interrupted Time Series			Difference-in-differences		
	(1)	(2)	(3)	(4)	(5)	(6)
Policy effect	0.736***	0.606**	0.730**	0.811**	1.019**	0.975**
	(0.177)	(0.250)	(0.307)	(0.230)	(0.305)	(0.347)
Control variables						
Month dummies	Yes	Yes	Yes	Yes	Yes	Yes
Patients casemix		Yes	Yes		Yes	Yes
Staff demographics			Yes			Yes
Total number of observations (week/ward)	463	463	463	331	331	331

*Note:* *<0.1, **<0.05, ***<0.01. Standard errors (in parentheses) are clustered at ward level. Regressions include patient casemix indicated by the total HoNOS score, the psychotic supercluster and number of patients per month, and staff demographics are indicated by the average staff age, percentage of female staff, percentage of white staff, and the percentage of registered nurses.

The test by means of a regression discontinuity framework shows no evidence of changes in the trend of covariates included in the analysis before and after the introduction of the 12-hour shifts, meaning that the policy has affected sickness absences, the outcome variable, but not the control variables. Estimation results of this test are reported in Appendix, Table A1.

Regarding the Interrupted Time Series specification, a test between fixed and random effects using the robust Hausman test (χ2 = 114.58) finds in favour of the correlated random effect framework (Wooldridge, 2010). The Interrupted Time Series estimation reported in the results section corresponds to the fixed effect panel data estimation, which is the preferred estimation.

### 3.3 Robustness checks

The series of checks performed suggest that our estimates are robust. A discussion of the results can be found in the Appendix.

## 4 Discussion

This study reports an evaluation of the impact of extending mental health nurses and health care assistants’ shifts from 8 to 12 hours. In particular, it explores the association between longer shifts and sickness absence, a widely used outcome within occupational health research, by comparing staff sickness absence rates before and after the introduction of a 12-hour shift pattern by means of Interrupted Time Series and Differences-in-Differences estimation approaches. The change in shift patterns from 8 to 12-hour shifts had a negative impact on 7-day sickness absence which increased between 0.73% according to Interrupted Time Series analysis and 0.98% according to the Difference-in-Differences estimation. All estimates are statistically significant at 95% confidence levels and robust to various sensitivity checks. The results are far from negligible as in a typical ward where there are 30 members of staff working full time (30 full-time equivalents (FTEs)), this would lead to approximately 12 hours of sickness absence per week – i.e. sickness absences will be increased by a complete shift per week, per ward as result of the new shift regime. These results are consistent with findings from the only previous study from Dall'Ora et al. (2019) examining general healthcare staff, who found that if a shift was scheduled to be 12-hours long, it would be 1.18 times more likely that staff would miss a shift when compared to an 8-hour shift schedule. The odds increase to 1.24 when long sickness absences are also included in the sample.

This study makes three key contributions: (i) This is the first study to analyse the impact of 12-hour shifts on sickness absences for mental health wards in England. The work from Dall'Ora et al. (2019) is the only previous study on sickness absence, however their data came from inpatient general adult wards. As previously stated, nursing mental health patients can be physically and emotionally more demanding than nursing patients with physical health problems. The former often can be impulsive, unpredictable, aggressive and can also often suffer physical health co-morbidities. Therefore, the extension to 12-hour shifts in mental health wards might lead to worse staff outcomes than those observed for general inpatient wards. (ii) The study uses longitudinal administrative data of all staff members from the sample wards, moving away from self-reported cross-sectional survey data often used to evaluate the impact of shift work on employee wellbeing. Jenkins and Elliott (2004) find that studies with self-reported questionnaires show inflation in reported stress. The switch to 12-hour shifts in this sample was compulsory, thus overcoming limitations of self-selection from previous studies where the policy has been optional. Furthermore, in this study all the wards are observed before and after the policy, allowing an unbiased estimation of the effect of the extended hours. This contrasts with Dall'Ora et al. (2019) who compare outcomes of two groups of individuals (often working in different wards) who are exposed to two (or more) different shifts patterns. In addition, the estimation strategy in this study accounts for a comprehensive set of covariates that control for contextual factors. Previous studies fail to report in sufficient detail contextual factors, such as staff skill mix or patient to nurse ratio, that are important determinants of the impact of different shift patterns (Harris et al., 2015). (iii) Finally, the application of Interrupted Time Series and Difference-in-Differences approaches is a methodological innovation in this context since it allows the separation of the effect of the 12-hour shifts on sickness absences from other confounders by using two different types of control groups. To our knowledge, this is the first study to use causal inference in the estimation of the impact of 12-hour shifts on sickness absences for mental health staff.

While this study makes important contributions, there are also limitations in terms of the generalisability of these findings which are limited since only a small number of wards were analysed, all of them belonging to the same hospital provider and therefore they might be different in some respects to other wards in other hospitals. It is important to note that as Reid et al. (1991) point out nurses’ behaviours, in this case short term sickness absence, might not be independent of their attitude towards the shift patterns they were working. With the data available, we are not able to disentangle if some of the observed sickness absences might be the result of a protest against 12-hour shifts rather than linked to health problems associated with the longer shifts. There is a small chance that there might be some unobserved factors affecting sickness absence that have not been accounted for. The latter is a common limitation of observational studies. Further, while our sickness absence data was available at the individual level, we did not have individual identifiers to track staff, and examine their characteristics, over time and therefore had to aggregate our analyses to ward level.

Further research should look at the effect of 12-hour shifts over a longer period of time, at the individual level, and evaluate the long run effects of working longer shifts. Not only the length of the shifts can have negative effects on staff sickness absences, but also how those shifts are distributed throughout the week. Ohayon et al. (2002) found that staff members from a Danish psychiatric hospital working on rotating day-night shifts were more likely to take sick leave when compared with those in the daytime group. Bourbonnais et al. (1992) found similar results for nurses from seven hospitals in Quebec. Ball et al. (2015) highlight the difficulties in assessing the effect of 12-hour shifts with little information about the practical ways the system is operated, i.e. how many shifts are worked in a row, number and lengths of breaks, etc. The data in this study does not include information on health care worker shift types or patterns and hence it is no possible to identify their effect on sickness absences. Further information on the distribution of shifts is needed to shed light on this issue.

In addition, most research evaluating the effect of longer shifts only focuses on staff acceptability and work-life balance (Harris et al., 2015) and few studies evaluate the effects on patient safety and experience (Griffiths et al., 2014). Future research should put more emphasis on patient measures as well as including an analysis of the cost-effectiveness of longer shifts. Therefore, further evidence on the costs and a wider set of outcomes on both patients and healthcare workers of a 12-hour shift system is needed to allow managers and policymakers a complete assessment of the policy.

Moreover, the data used in this study is at an aggregate level, where sickness absences as well as control variables are captured at week/ward level. Longer shifts entail different risks and benefits for different staff at different times (Ball et al., 2015), e.g. there might be greater risks for the older person working long shifts (Chudleigh et al., 2005). Further research could consider individual data to disentangle which demographic groups are more likely to suffer the negative effects of 12-hour shifts. According to Harris et al. (2015) the success of the 12-hour shift will ultimately be dependent upon the support and cooperation of the staff involved. A qualitative study on the effects of 12-hours shifts on the same mental health hospital from this study, (Suter et al., 2019), shows that, whilst a 12-hour shift was received positively by some staff as it offered them flexibility, when implemented as a mandatory work pattern, the element of employee choice is eliminated leading to dissatisfaction and disinterment amongst others. If staff have negative views and negative work outcomes persist in the long-run then the policy is likely to fail.

## 5 Conclusion

Our findings suggest that the switch to 12-hour shifts has increased sickness absences and, whilst wider aspects of the policy change are important, including alternative outcomes and overall cost-effectiveness, if only sickness absences were taken into account, hospital managers should consider reverting to 8-hour shifts. Our analysis is relevant to other hospitals within England, and internationally, which are increasingly moving towards these shift patterns.

## Credit Author Statement

IRS, MAM, RJ, and MC: contributed to the design and implementation of the research and to the analysis of the results. MAM: carried out the data analysis. IRS: wrote the initial draft with help from MAM. RJ: Supervised the study. JS, TK, RJ, IRS and MC: worked on the funding acquisition. All authors provided critical feedback and worked in the writing, reviewing and editing process.

## Ethical Approval

This study received ethics approval from the Health Research Authority - 18-HRA-0454.

## Funding sources

This work is part funded by the Wellcome Trust [ref 204829] through the Centre for Future (CFH) at the University of York.

## Conflict of interest

None.

## Figures and Tables

**Table A.1:** Discontinuity of covariates at the time of introduction of the policy.

	RD Effect	Robust P-value
Casemix adjustment monthly average HoNOS score	-0.47	0.366
Supercluster group 2 (Perc. x 100)	0.919	0.944
Number of patients per month	-0.28	0.914
Number of staff per month	-0.147	0.987
Female staff (Perc. x 100)	0.403	0.983
Staff ethnicity white (Perc. x 100)	-0.16	0.913
Staff average age (in years)	-0.086	0.968
Registered Nurses (Perc. x 100)	0.468	0.757

*Note:* Test of discontinuity of covariates at the time of introduction of the policy by means of the `rdrobust’ Stata command. The first column, RD effect, reports the regression discontinuity coefficient for each covariate. The second column reports the Robust P-value. None of the RD effects are statistically significant.

**Fig. A.1:**
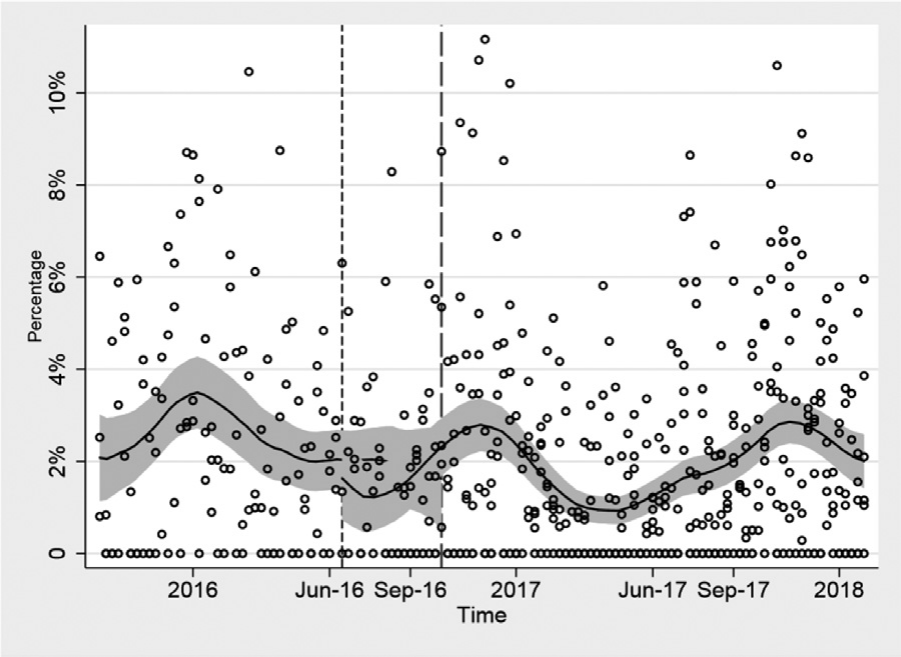
Placebo test, introduction of the policy one year before. The solid horizontal line is the local polynomial smooth of the dotted scatter values using a triangle kernel function; the shaded area represents the 95% confidence interval around it and the vertical lines indicate the timing of the introduction of the policy.

**Table A.2:** Main estimation results for the Interrupted Time Series (ITS) (1) and Difference-in-Differences (DID) (3) models and estimation results for the ITS (2) and DID placebo tests (4).

	ITS		DID	
	(1)	(2)	(3)	(4)
Policy effect (ITS)				
Policy effect (T)	0.729**	-0.684*		
	(0.307)	(0.390)		
Trend	0.016***	0.024		
	(0.004)	(0.041)		
Iteration trend x T	-0.009	-0.03		
	(0.016)	(0.041)		
Policy effect (diff-diff)				
Policy effect (iteration time of policy x treated wards)			0.975**	0.350
			(0.347)	(1.319)
Time of the policy			-0.496	0.612
			(0.336)	(1.319)
Treated wards			0.595	-17.979
			(0.457)	(9.299)
Patient control variables				
Casemix adjustment monthly average HoNOS score	0.046**	0.064***	0.066	-0.442
	(0.022)	(0.018)	(0.035)	(0.355)
Supercluster group 2 (Perc. x 100)	-0.031***	-0.030***	-2.720***	15.263
	(0.008)	(0.008)	(0.629)	(8.252)
Number of patients per month	0.01	0.01	0.042	-0.518***
	(0.029)	(0.037)	(0.021)	(0.007)
Staff control variables				
Staff average age (in years)	0.136	0.2	-0.126**	-0.471
	(0.181)	(0.174)	(0.048)	(0.342)
Female staff (Perc. x 100)	0.001	0.004	3.897**	-32.202**
	(0.030)	(0.032)	(1.319)	(3.673)
Staff ethnicity white (Perc. x 100)	-0.001	-0.024	-1.746	-230.413*
	(0.043)	(0.045)	(2.778)	(74.593)
Registered Nurses (Perc. x 100)	0	-0.005	0.859	34.375**
	(0.038)	(0.043)	(2.191)	(4.567)
Number of staff	0.038	0.04	0.025*	-0.003
	(0.024)	(0.026)	(0.010)	(0.025)
Time variables				
Month dummies	Yes	Yes	Yes	Yes
Total number of observations (week/ward)	463	463	331	45

*Note:* *<0.1, **<0.05, ***<0.01. Standard errors (in parentheses) are clustered at ward level. Columns (1) and (3) correspond to main results in the fixed effect model and difference-in-difference, respectively. Columns (2) and (4) show the results for the placebo test, that assumes the policy was introduced one year before on the same month and day, for the ITS and the DID models respective.

**Table A.3:** Robustness check Interrupted Time Series (ITS) specification.

	(1)	(2)	(3)	(4)	(5)
Policy effect					
Policy effect (T)	0.813**	0.730**	0.706**	0.042	0.900**
	(0.324)	(0.298)	(0.302)	(0.241)	(0.446)
Trend	0.013**	0.069	0.188	0.006	-0.022**
	(0.006)	(0.058)	(1.123)	(0.007)	(0.008)
Iteration trend x T	-0.006	-0.013	-0.015	-0.012	0.004
	(0.018)	(0.024)	(0.024)	(0.011)	(0.010)
Patient control variables					
Casemix adjustment monthly average HoNOS score	0.047	0.042	0.042	-0.031	0.101***
	(0.030)	(0.027)	(0.027)	(0.025)	(0.039)
Supercluster group 2 (Perc. x 100)	-0.020***	-0.031***	-0.031***	-0.016**	-0.037***
	(0.002)	(0.009)	(0.009)	(0.004)	(0.011)
					
Number of patients per month	0.036*	0.006	0.001	-0.016	0.051
	(0.019)	(0.038)	(0.042)	(0.019)	(0.045)
Staff control variables					
Staff average age (in years)	-0.177***	0.145	0.161	0.184	0.031
	(0.024)	(0.177)	(0.176)	(0.117)	(0.223)
Female staff (Perc. x 100)	0.014*	0	0.001	0.001	0.023
	(0.008)	(0.027)	(0.027)	(0.025)	(0.050)
Staff ethnicity white (Perc. x 100)	-0.025	-0.005	-0.006	-0.042*	0.035
	(0.029)	(0.038)	(0.039)	(0.021)	(0.073)
Registered Nurses (Perc. x 100)	-0.025	-0.003	-0.002	-0.028*	0.007
	(0.018)	(0.034)	(0.038)	(0.014)	(0.057)
Number of staff	0.01	0.038*	0.040*	0.029	0.036
	(0.016)	(0.023)	(0.024)	(0.022)	(0.041)
					
Time variables					
Month dummies	Yes	Yes	Yes	Yes	Yes
Year dummies		Yes	Yes		
Week dummies			Yes		
Total number of observations (week/ward)	463	463	463	463	463

*Note:* *<0.1, **<0.05, ***<0.01. Standard errors (in parentheses) are clustered at ward level. Column (1) shows the estimation results for the pooled OLS; Column(2) shows the estimates for fixed effect with yearly dummies and (3) with the addition of weekly dummies Column (4)shows results for sickness absence up to two days and (5) up to 14 days.

## Appendix

### Robustness Checks

Regarding the robustness check number one (i.e the placebo test), Fig. A1 shows that this arbitrary policy does not seem to change the percentage of sickness absences per ward/week. Moreover, Table A2 presents the regression results for the Interrupted Time Series and Difference-in-Differences for the placebo test and no effect on the rate of sickness absence is found, suggesting that the findings are robust.

Regarding the robustness checks two, using pooled Ordinary Least Squares instead of fixed effects panel data, and three, using sickness absences of 14 days and 2 days, all estimations with the exception of sickness absences up to 2 days provide estimates with confidence intervals that contain the main results presented (details in Table A3), suggesting results are driven by sickness absences from 3 to 7 days. Robustness check number four, where the policy implementation date is moved artificially six weeks prior and six weeks post the actual date, shows no evidence for anticipatory effects up to two weeks before the policy; however a consistent and increasing effect was found from the introduction of the policy and up to three weeks after, with the greatest effect found in the third week. The latter suggests the existence of some delayed effects which are expected, since the effects on sickness absence due to the introduction of the policy are more likely to increase in the medium/long run when tiredness, stress and burnout accumulates due to the ongoing experience of longer shifts. Results from robustness check (iv) are available upon request.
